# MRI Assessment of the Early Disc Degeneration Two Levels above Fused Lumbar Spine Segment: A Comparison after Unilateral and Bilateral Transforaminal Lumbar Interbody Fusion (TLIF) Procedure

**DOI:** 10.3390/jcm11143952

**Published:** 2022-07-07

**Authors:** Milka Kljaic Dujic, Gregor Recnik, Milko Milcic, Eva Bosnjak, Mitja Rupreht

**Affiliations:** Department of Radiology, University Medical Centre Maribor, 2000 Maribor, Slovenia; gregorrecnik@yahoo.com (G.R.); milko9464@hotmail.com (M.M.); eva.bosnjak1@gmail.com (E.B.); mitja.rupreht@ukc-mb.si (M.R.)

**Keywords:** spine, intervertebral disc degeneration, surgical procedure, orthopedic procedure, surgical fixation, magnetic resonance imaging

## Abstract

Background: Adjacent segment degeneration (ASD) has become a great concern as a late complication in patients following fusion spine surgery with a potential need for revision surgery. Segments above the fused spine have higher mobility and they are especially prone to accelerated disc degeneration. The purpose of our study is to investigate early disc degenerative changes two levels above a surgically fused lumbar spine segment and to compare MRI analyses after unilateral and bilateral TLIF procedures. Methods: A total of 117 patients were included in this cross-sectional retrospective single center study (after bilateral TLIF surgery: *n* = 91, and after unilateral TLIF: *n* = 26). In both groups, the average patient age was similar: 62.84 years (SD = 12.53) in the unilateral TLIF group and 60.67 years (SD = 11.89) in the bilateral TLIF group. On average, MRI was performed 2.5 years after surgery (SD = 2.09). The modified eight-level Pfirrmann grading system was used for the assessment of disc degeneration severity. Descriptive statistics and the Mann–Whitney test were used to show differences in the Pfirrmann grades regarding the after-surgery period and the patient age. The Wilcoxon signed-rank test results were used to display differences in the Pfirrmann grades before and after surgery. Results: The comparison of mean values, regardless of the type of surgery, shows that this mean value is on average higher in the first segment adjacent to the fused spine segment. The assessment of the intervertebral disc structure in BIL TLIF is higher in both the first and the second segment. Early disc degeneration progression is subtle yet detectable (UNI TLIF 9.28% vs. BIL TLIF 16.74%). The assessment of the intervertebral disc structure is on average lower in patients aged less than 50 years at time of surgery compared with patients aged more than 50 years in UNI TLIF, and higher in the BIL TLIF group, for both the first and the second segment. Conclusion: Patients who had undergone unilateral TLIF fusion surgery have a lower rate of early disc degenerative changes. Considering a significantly higher rate of progressive disc degenerative changes in the elderly with bilateral fusion surgery, extra caution is required in the selection of appropriate surgical technique.

## 1. Introduction

Lumbar degenerative disc diseases (DDD) account for the majority of patients with lower back pain, which sometimes still remains difficult to diagnose [1,2]. The transforaminal lumbar interbody fusion (TLIF) is an effective surgical method that is commonly used for treating DDD, providing clinical improvement to patients [2,3].

The bilateral pedicle screw fixation (BPSF) is acknowledged as a standard procedure demonstrating increased intervertebral fusion rates. Additionally, it contributes to strong stability between vertebrae and can maintain the height of the segment [4]. The unilateral pedicle screw fixation (UPSF) tends to be less invasive compared with BPSF. In addition, the operative time is shorter, and patients present with a reduced amount of blood loss during surgery. Furthermore, with this type of fixation, fewer posterior structures are damaged in the process. Additionally, the stiffness that follows BPSF may play a role in increasing stress at intervertebral segments that lie adjacent to the fused segment. This could be, among other factors, another reason for adjacent segment pathology [5,6]. Adjacent segment degeneration (ASD) may potentially occur as a long-term complication after undergoing a spinal fusion surgery [7]. There are many risk factors for ASD following lumbar fusion surgery; however, some of them remain controversial [8]. The aim of this study is to radiologically compare cranial adjacent intervertebral discs on MRI studies of patients that have undergone unilateral versus bilateral transforaminal lumbar interbody fusion (TLIF). The purpose is to compare disc degeneration two segments above the fused spine and the mean values between UNI TLIF and BIL TLIF. In addition, our goal is to compare disc degeneration grades in the same patients before surgery and 2.5 years after surgery and to establish whether it is possible to detect such early disc changes using MRI and how these changes are displayed in percentage. The following analysis should also show disc changes after UNI TLIF and BIL TLIF according to patient age (younger than 50 years and older than 50 years) and the differences between two surgical techniques according to time after surgery (5 years before surgery and 5 years after surgery). Adjacent segment degeneration has become a great concern in patients after fusion surgery procedure. Due to higher mobility, cranial segments are especially prone to long-term progressive disc degeneration and, consequently, revision surgery. Data about the progression of cranial adjacent segment degeneration and comparison between UNI TLIF and BIL TFLIF are still insufficient.

## 2. Methods

A total of 117 patients were included in this cross-sectional retrospective single center study (after bilateral TLIF surgery: *n* = 91, and after unilateral TLIF: *n* = 26). All patients had undergone TLIF fusion surgery between L1-5 lumbar spine segments. The inclusion criteria for patient selection were subjects after one-level minimally invasive unilateral TLIF or one/multi-level bilateral TLIF. In the first group of those who had undergone unilateral TLIF, there was only a one-level fused lumbar segment. In the second group with bilateral TLIF, there were one-level fusions, two-levels fusions, and three-levels fusions. The exclusion criteria were patients who had undergone revision surgery or a different surgical technique (anterior procedure ATLIF, posterior PTLIF, or extreme lateral approach XLIF) and patients after surgical procedures due to tumor, vertebral fracture, or spinal infection.

The study has been approved by the Institutional and National Ethics Committee (No 0120-569/2015-2). Magnetic resonance images (MRI) measurements were conducted according to a standard protocol for lumbosacral spine at L1-S1 levels in a supine patient position. Pre- and postoperative lumbar spine MRI was acquired using a 1.5T MR Scanner (Magnetom Aera, Siemens Healthineerse AG, Erlangen, Germany). Patients underwent MRI on average 2.5 years after surgery (SD = 2.09). The modified Pfirrmann grading system was used to assess the severity of disc degeneration. This grading system is based on disc structure (the signal intensity from the nucleus and the differentiation between the inner and outer fibers of the annulus at the posterior aspect of the disc) and the preservation of disc height. This eight-level modified grading system is easy to use as it provides enough discriminatory information—especially in assessing elderly patients’ MRIs—and solid inter- and intraobserver reliability [9]. The data analyses were performed using SPSS statistics software version 25 (SPSS Inc., Chicago, IL, USA). Means and standard deviations were calculated. Descriptive statistics and the Mann–Whitney test were used to show differences in Pfirrmann grades regarding the time after surgery and patient age. The Wilcoxon signed-rank test results were used to display differences in the Pfirrmann grades before and after the surgical procedure.

## 3. Results

A total of 117 patients were included in this study (after bilateral TLIF surgery: *n* = 91, and after unilateral TLIF: *n* = 26). There were 44 female and 73 male patients (UNI TLIF 13 M, 12 F; BIL TLIF 31M, 61F). UNI TLIF were all one-level surgical procedures, while BIL TLIF were one- and multi-level surgical procedures (55 one-level, 25 two-levels, and 9 three-levels). An analysis between these bilateral subgroups has not been made because the focus was on the comparison between UNI TLIF vs. BIL TLIF. For analysis of early disc degeneration changes, 55 patients were qualified (on average 2.4 years after surgery, deviation within 6 months). The comparison of mean values regardless of the type of surgery for the assessment of the intervertebral disc structure shows that this mean value is on average higher in the first segment (adjacent to the fused segment) than the second segment (two levels above the fused spine segment) (Table 1). The assessment of the intervertebral disc structure in BIL TLIF is higher both in the first and the second segment regarding the UNI TLIF.

**Figure 1:**
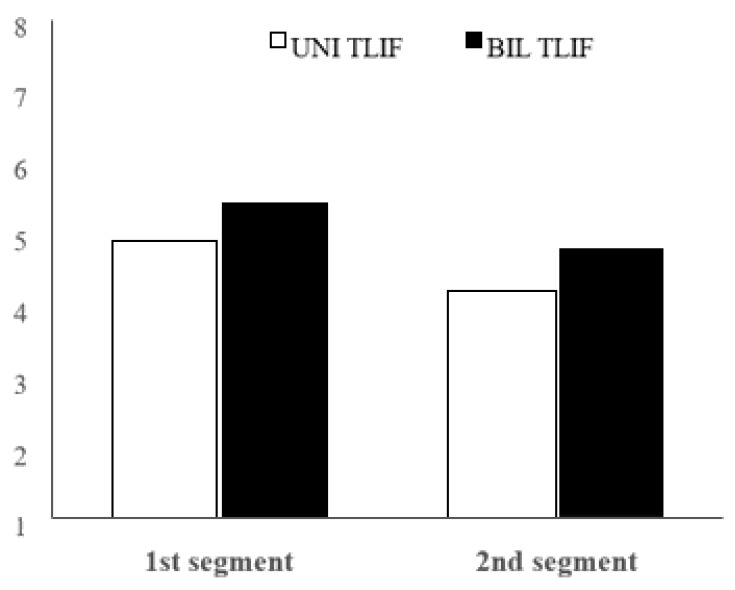
Comparison of the mean intervertebral disc structure assessment between UNI TLIF (unilateral transforaminal lumbar interbody fusion) and BIL TLIF (bilateral TLIF) on the 1st and 2nd segment.

There are statistically significant differences (U = 834.0 *p* = 0.042) in the assessment of the intervertebral disc structure between the UNI TLIF and the BIL TLIF in the second segment. The assessment after the BIL TLIF is statistically significantly higher than the assessment after the UNI TLIF (Figure 1).

**Figure 2:**
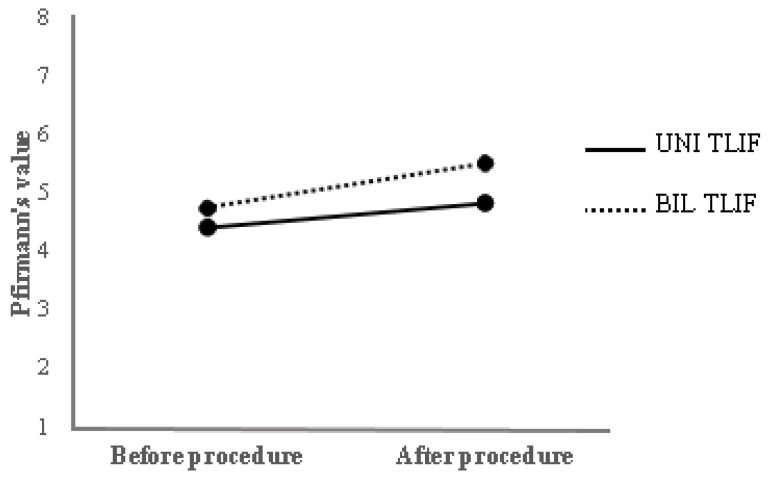
The comparison of an average Pfirrmann grade between UNI TLIF and BIL TLIF before and after surgery one level above fused spine segment.

Patients with available MRI data before and after surgery were included in the next analysis (Figure 2). A comparison has been made between the Pfirrmann grades before and after the surgical procedure in the same patients (Table 2). Pfirrmann grades after the UNI TLIF procedure were 9.28% higher than before the operation. The same grades after the BIL TLIF procedure were 16.74% higher compared with grades before the surgical procedure. 

**Figure 3:**
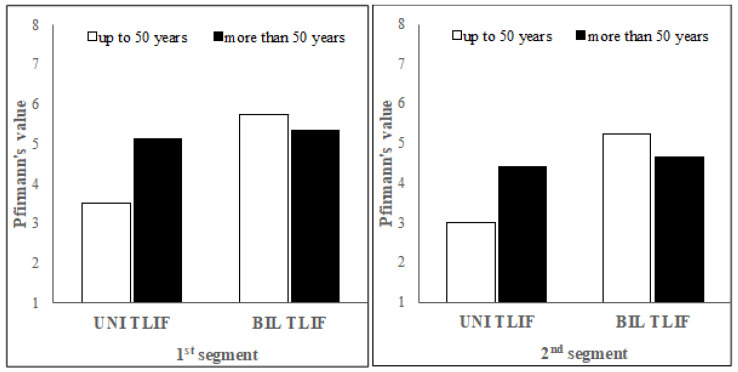
Comparison of the differences in Pfirrmann grades in patients up to 50 years old and in patients more than 50 years old.

The following analysis considering age shows patients who underwent UNI TLIF surgery who were aged between 32 and 84 at the time of surgery, on average 62.84 years (SD = 12.536) (Table 3). The patients who underwent BIL TLIF surgery were aged between 38 and 85 at the time of surgery, on average 60.67 years (SD = 11.894). The assessment of the intervertebral disc structure is on average lower in patients aged less than 50 years at the time of surgery compared with those who were aged more than 50 years in the UNI TLIF group, and on average higher in the BIL TLIF surgery group for both the first and the second segment (Figure 3).

The BIL TLIF patients show differences in the assessment of the intervertebral disc structure between the two age groups; however, the differences are not statistically significant.

The next analysis revealed that patients who underwent MRI evaluation more than 5 years after surgery had a higher average Pfirrmann grade than patients who underwent surgery less than 5 years previously (Table 4). This also held true for patients who underwent BIL TLIF on the first and the second segment, and in patients who underwent UNI TLIF on the first segment. The opposite held true for patients who underwent UNI TLIF on the second segment.

## 4. Discussion

Magnetic resonance imaging is a sensitive and reliable diagnostic tool that enables visualization even for early structural and morphological intervertebral disc changes [10,11,12,13,14]. Most of the previous clinical studies have compared postoperative surgical complications and clinical outcomes between UNI TLIF and BILL TLIF. Postoperative advantages of the UNI TLIF, such as reduced intraoperative bleeding, shorter hospital stays, and quicker rehabilitation rates are very well documented [15,16,17,18,19,20,21]. In addition, surgical success rates and clinical outcomes in both UNI TLIF and BIL TLIF, have been documented [22,23,24,25,26,27,28,29,30]. There have not been many studies comparing early disc degenerative changes on MRI and the progression of disc degeneration of cranial adjacent segments.

In their ten-year follow-up study, Kim and authors [31] have shown a lower rate of radiological adjacent segment degeneration and clinical outcome in the unilateral pedicle screw fixation group (UPSF 55.9% vs. BPSF 72.7%). Authors concluded that UPSF, in comparison to BPSF, can contribute to a reduction in degeneration of cephalad adjacent segments. The results of our analysis are similar to those obtained in their study, except, in our study, degenerative disc changes are detectable and changes are documented earlier (2.5 years after surgery).

In their 15-year follow-up study, Maruenda and authors [32] have shown how important adjacent segment degeneration (ASD) after circumferential lumbar fusion is. In a period from 2 to 15 years, patients’ outcomes have worsened significantly. Revision surgery of symptomatic ASD was highly dependent on age and the number of fused levels. At a 2-year follow-up, the ODI score was significantly lower. Five years after surgery, about 10% of patients required reoperation due to symptomatic ASD. After 10 years, 24.6% of patients required reoperation, and after 15 years there was an even higher number of patients who underwent reintervention (37.5%). As the authors concluded, this high rate of ASD and reinterventions questions the long-term reliability of this surgical technique.

In their meta-analysis, Drysch and authors [26] suggest that the most effective surgical treatment has not been established for ASD. There was a high variability in the clinical improvement following lumbar fusion and revision surgery, between 4.5% and 23.1%. In conclusion, the literature is limited on the optimal treatment options for ASD patients.

Another meta-analysis study by Muthu and authors [33], compared the safety of unilateral and bilateral instrumented fusion in second-level degenerative disorders. Unilateral surgical technique provides immediate safety, reduced intraoperative bleeding, and a shorter hospital stay and rehabilitation. They concluded that there is still a lack of high-quality evidence of complications, such as cage subsidence and ASD.

In summary, our results confirmed that the mean values of disc degeneration, irrespective of the surgical method, are higher in the first segment (adjacent to the fused segment) compared with the second segment (two levels above the fused spine segment). In comparison with the UNI TLIF, the assessment of the intervertebral disc structure in BIL TLIF is higher in both the first and the second segment. This is in line with expectations. In the first group—patients who had undergone unilateral TLIF—there were only one-level fused lumbar segments. In the second group with bilateral TLIF, there were 55 one-level fusions, 25 two-levels fusions, and 9 three-levels fusions. We have not compared these bilateral subgroups, because the focus was on the comparison of UNI TLIF vs. BIL TLIF. Limitations may exist, in particular, in the last subgroup due to a small sample size for statistical analyses. It would be meaningful to compare these in a further analysis using a larger sample size. Early disc degeneration progression is detectable and faster after BIL TLIF, as expected. Differences in disc degeneration in the elderly group (patients over 50) were greatest after UNI TLIF at the first and second segments, but not statistically significant after BIL TLIF. We did expect the differences to be statistically significant after BIL TLIF. Furthermore, the results of the 5-year follow-up after surgery were inconsistent, especially for the assessment of the second segment, which could be attributed to the statistically smaller patient sample size in this group. The study’s limitations included a smaller sample size for subjects 5 years following surgery. Despite a small sample size of subjects and a late follow-up, we expected that the degenerative changes would become more visible, detectable, and distinct over time (with even major differences). However, the results were somewhat conflicting, especially for the second segment lacking an unambiguous conclusion. In summary, disc degeneration due to higher stress in cranial segments is hardly avoidable after fusion surgery; however, UNI TLIF can contribute to reducing disc degenerative changes.

## 5. Conclusions

Patients who had undergone unilateral TLIF fusion surgery had a lower rate of disc degenerative changes. Considering the significantly higher rate of progressive disc degenerative changes in the elderly with bilateral TLIF fusion surgery, extra caution is required in the selection of appropriate surgical technique.

## Figures and Tables

**Table 1:** Descriptive statistics and Mann–Whitney test results for the differences in Pfirrmann grades. A comparison between unilateral and bilateral transforaminal lumbar interbody fusion procedures. Mean, standard deviation, statistically significant differences and *p*-value were calculated.

	UNI TLIF (*n* = 25)		BIL TLIF (*n* = 90)		Mann–Whitney Test	
	M	SD	M	SD	U	*p*
1st segment	4.88	1.130	5.42	1.390	889.5	0.102
2nd segment	4.20	1.000	4.79	1.276	834.0	0.042

**Table 2:** Descriptive statistics and Wilcoxon signed-rank test results for the differences in Pfirrmann grades before and after procedure.

	Before Procedure		After Procedure			Wilcoxon Signed-Rank Test	
	M	SD	M	SD	*n*	Z	*p*
UNI TLIF	4.42	1.084	4.83	1.115	12	−2.236	0.025
BIL TLIF	4.74	1.482	5.51	1.420	43	−4.412	0.000

**Table 3:** Descriptive statistics and Mann–Whitney test results for the differences in Pfirrmann grades for patients up to 50 years old and patients more than 50 years old.

	Up to 50 Years			More than 50 Years			Mann–Whitney Test	
	*n*	M	SD	*n*	M	SD	U	*p*
1st segment								
UNI TLIF	4	3.50	0.577	21	5.14	1.014	8.0	0.009
BIL TLIF	18	5.72	1.406	72	5.35	1.386	544.0	0.284
2nd segment								
UNI TLIF	4	3.00	0.816	21	4.43	0.870	10.5	0.013
BIL TLIF	18	5.22	1.309	72	4.68	1.254	516.5	0.173

Statistically significant differences in the average assessment of the intervertebral disc structure between patients younger than 50 years and those older than 50 years are present at UNI TLIF on both segments; on the 1st segment (U = 8.0 *p* = 0.009) and on the 2nd segment (U = 10.5 *p* = 0.013).

**Table 4:** Descriptive statistics and Mann–Whitney test results for the differences in Pfirrmann grades regarding the time after surgical procedure (up to 5 years and more than 5 years).

	Up to 5 Years			More than 5 Years			Mann–Whitney Test	
	*n*	M	SD	*n*	M	SD	U	*p*
1st segment								
UNI TLIF	23	4.78	1.085	2	6.00	1.414	11.0	0.211
BIL TLIF	83	5.41	1.362	7	5.57	1.813	275.0	0.811
2nd segment								
UNI TLIF	23	4.22	0.998	2	4.00	1.414	20.5	0.790
BIL TLIF	83	4.77	1.262	7	5.00	1.528	236.5	0.403
